# Case report: Occult *Listeria monocytogenes* invasion leading to prosthetic hip joint infection in a patient with rheumatoid arthritis taking tofacitinib

**DOI:** 10.3389/fmed.2023.1322993

**Published:** 2024-01-09

**Authors:** Chaowen Deng, Kelvin Hei-Yeung Chiu, Nan Lou, Fanfan Xing

**Affiliations:** ^1^Department of Clinical Microbiology and Infection Control, The University of Hong Kong-Shenzhen Hospital, Shenzhen, Guangdong, China; ^2^Department of Microbiology, Queen Mary Hospital, Hong Kong, Hong Kong SAR, China; ^3^Department of Orthopedics, The University of Hong Kong-Shenzhen Hospital, Shenzhen, Guangdong, China

**Keywords:** *Listeria monocytogenes*, prosthetic joint infection, rheumatoid arthritis, tofacitinib, case report

## Abstract

It has been suggested that targeted therapy may potentially increase the risk of listeriosis. However, no reported cases of *Listeria monocytogenes* prosthetic joint infection have been documented during Janus Kinase (JAK) pathway inhibitor use. Herein, we present a 70-year-old female with rheumatoid arthritis who had undergone bilateral hip joint replacement and subsequently developed *Listeria monocytogenes* prosthetic joint infection following tofacitinib therapy. We suggest that the use of tofacitinib may potentially heighten susceptibility to listeriosis in patients afflicted with rheumatoid arthritis.

## Introduction

Listeriosis is a serious foodborne illness that is caused by *Listeria monocytogenes* or rarely *Listeria ivanovii*. Patients are usually infected by consuming unpasteurized milk or dairy products, contaminated raw meat, and ready-to-eat food. In immunocompetent hosts, listeriosis usually presents as an acute self-limited gastroenteritis. However, in elderly, pregnant, and other immunocompromised patients, *Listeria monocytogenes* can cause invasive infections such as bacteremia, central nervous system infections, or focal infections (1). While infections of prosthetic joints caused by *L. monocytogenes* are rare, the incidence of this disease is increasing with the increase in number of joint replacements performed in the high-risk population (2).

Targeted therapy is a treatment approach that specifically targets cellular and molecular factors, and is widely used in the treatment of various autoimmune diseases, and solid organ and hematologic malignancies. Because the targets of this therapy are major regulators of the body’s immune system, they are typically associated with infection that requires specific types of immune responses to control. Various opportunistic infections have been reported in association with different types of targeted therapy, with tuberculosis and invasive fungal infections being the most commonly reported infections. Tofacitinib is a new small-molecule targeted drug for the treatment of autoimmune diseases such as rheumatoid arthritis and Sjogren’s syndrome. It belongs to the JAK pathway inhibitor and preferentially inhibits JAK3 and JAK1, and regulates immune responses by downregulating cytokines such as interleukin (IL)-2, 4, 7, 9, 15, and 21 (3). We used “*Listeria monocytogenes*” and “JAK pathway inhibitors “as search terms in the PubMed database and found no clinical reports of *Listeria* infections associated with the use of JAK pathway inhibitors.

Herein, we report a case of late-onset prosthetic left hip joint infection caused by *L. monocytogenes* in a patient with rheumatoid arthritis who received tofacitinib and propose for the first time that the use of JAK pathway inhibitors in patients with rheumatoid arthritis may also be a risk factor for *Listeria* infection.

## Case presentation

On April 11, 2022, a 70-year-old female was admitted to The University of Hong Kong-Shenzhen Hospital with 6 months of purulent discharge from the incision wound of left hip arthroplasty. The patient has a medical history spanning over two decades, which includes hypertension, diabetes mellitus, and rheumatoid arthritis. She received metoprolol and amlodipine besylate for blood pressure control and performed regular exercise and dietary control for her diabetes mellitus. Since 2012, she has been taking methotrexate, leflunomide, and hydroxychloroquine for the control of rheumatoid arthritis. In 2009, the patient underwent right hip arthroplasty at an external hospital due to bilateral hip osteoarthritis and avascular necrosis of the femoral head caused by long-term steroid use. Then in July 2020, left hip arthroplasty was performed at our hospital for developmental dysplasia of the left hip with secondary osteoarthritis.

In January 2021, the patient experienced pain in her metacarpophalangeal joints along with elevated inflammatory markers leading to the consideration of active rheumatoid arthritis. Her treatment was adjusted to include tofacitinib (5 mg orally once daily) and leflunomide (10 mg orally once daily). However, a distal fistula with exudation was developed at the incision site from the previous left hip arthroplasty in October 2021. Treatment with erythromycin ointment was ineffective, therefore she was referred to our hospital for further management.

**Figure 1:**
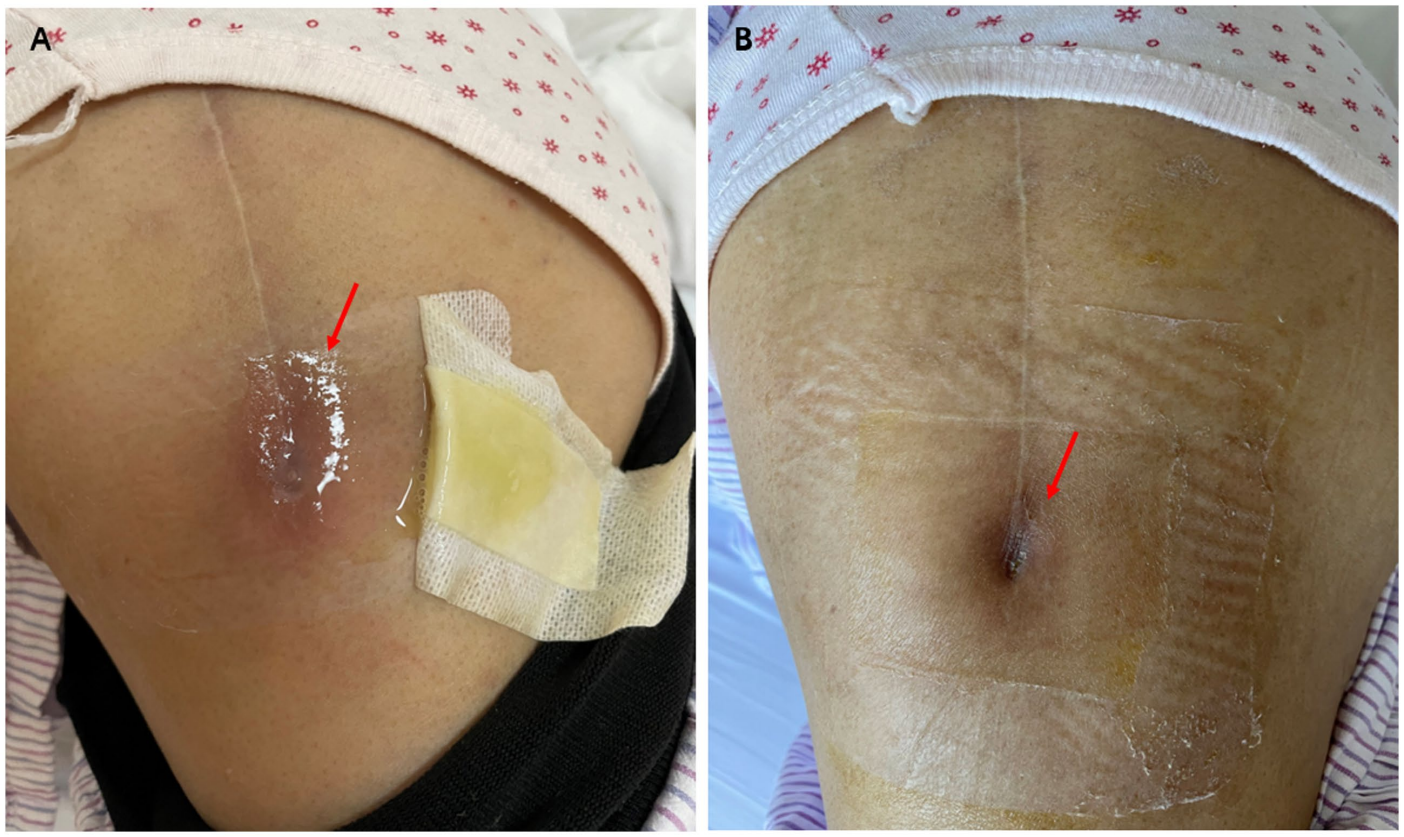
The fistula is shown before **(A)** and after **(B)** antibiotic treatment.

Upon admission, the patient had a body temperature of 36.8°C, a blood pressure of 116/74 mmHg, a pulse rate of 76 beats per minute, and a respiratory rate of 18 breaths per minute. Physical examination revealed no palpable superficial lymph nodes except a local fistula at the distal end of the incision site from her previous left hip surgery (Figure 1A). Transparent, clear, and viscous fluid was expressed from the fistula. No erythema or swelling was observed around the incision site, and there was no tenderness at the hip joint. Blood tests revealed a white blood cell count of 6.61 × 10^9^/L (normal range, 3.89–9.93 × 10^9^/L), with 61.6% neutrophils and 31.3% lymphocytes, a hemoglobin level of 115 g/L (normal range, 115–148 g/L), and a creatinine level of 150umol/L (normal range, 44–80umol/L). The erythrocyte sedimentation rate was elevated at 71 mm/h (normal range, 0–20 mm/h), while C-reactive protein levels were also increased to 12.47 mg/L (normal range, 0–5 mg/L). Other laboratory tests including liver function, procalcitonin, and rheumatoid factor showed no significant abnormalities.

**Figure 2:**
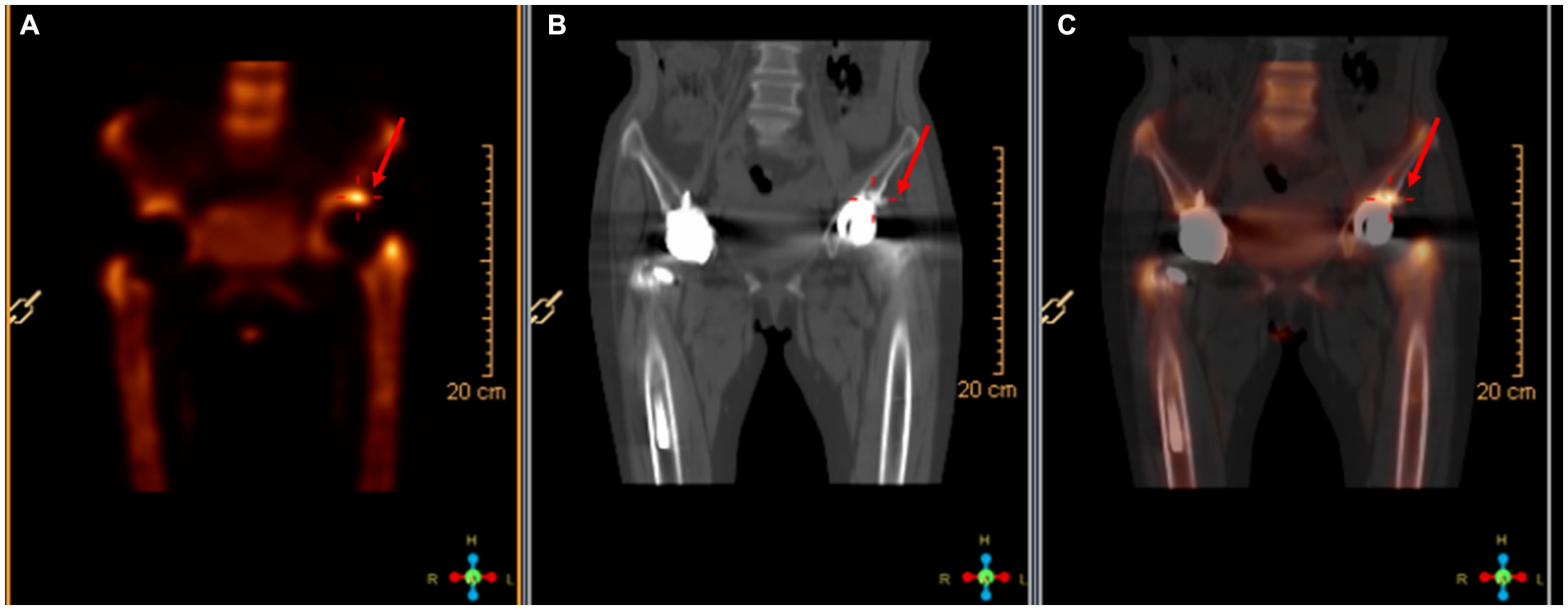
Radionuclide bone imaging. **(A)** The bone metabolism around the left femoral stem was increased. **(B)** The blood flow phase was normal. **(C)** The radioactive uptake in the blood pool phase was increased.

**Figure 3:**
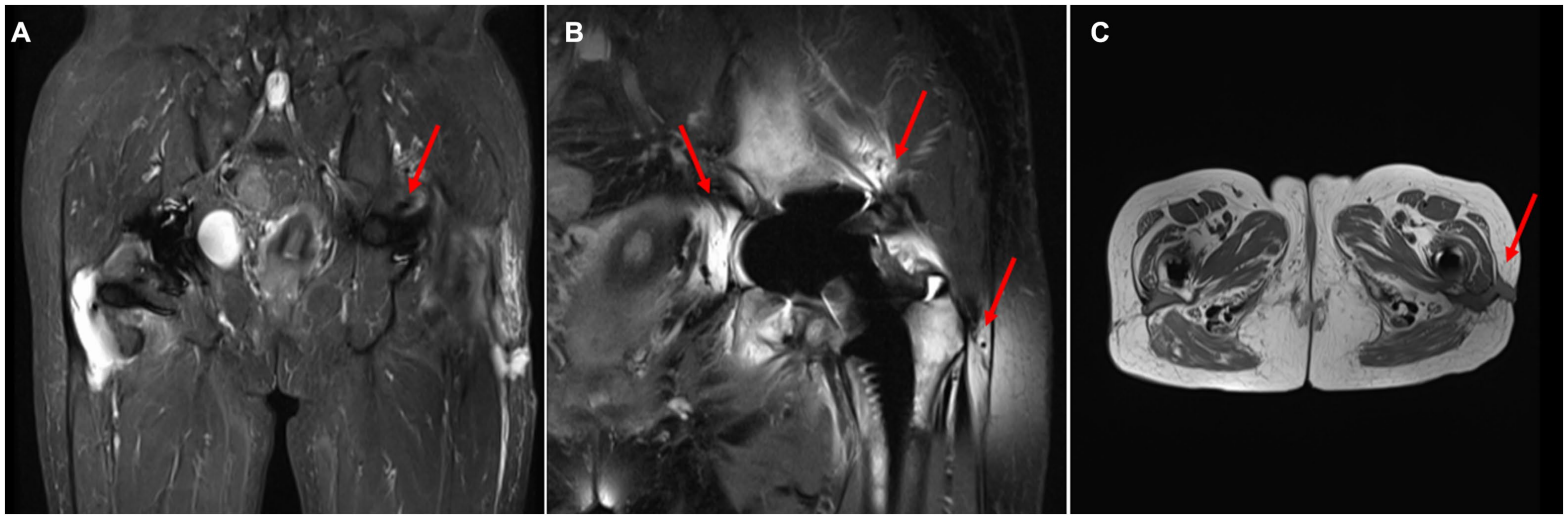
MRI of the hip joint. **(A)** Localized hyperintensity was found in the skin and fascia of the lateral thigh on the left side(T2). **(B)** The bone and muscle around the left upper femur were hyperintense(T2). **(C)** Subcutaneous abscess and fistula formed in the upper part of the left thigh.

On April 13, a sample of pus from the fistula was collected for aerobic and anaerobic bacteria culture. Gram stain of the specimen revealed a moderate number of leukocytes but no organisms were seen. After 2 days of incubation, scanty growth of coagulase-negative *Staphylococcus* and *Listeria monocytogenes* were identified. Additional pus was collected 4 days later, with pure growth of *Listeria monocytogenes* isolates. Two sets of blood cultures were taken with incubation for 14 days, and additional stool specimens were sent with enrichment using blood agar with aztreonam, but these results were unremarkable. On April 22, whole-body bone scintigraphy (Technetium 99 m-methyl diphosphonate) showed an increased bone metabolism around the stem of the left femoral prosthesis and an increased radionuclide uptake in the blood pool phase, suggesting postoperative chronic infectious lesions (Figure 2). On April 24, magnetic resonance imaging (MRI) of the left hip joint showed inflammatory exudation around the upper end of the femur and surrounding soft tissue, and a subcutaneous abscess with fistula formation in the upper thigh (Figure 3). Transthoracic echocardiography and cranial MRI were performed, but no significant abnormalities were found. Upon further history taking with a food questionnaire, the patient reported consumption of pasteurized milk stored in the refrigerator, and she developed mild gastroenteritis approximately 1 month before infection. It is suspected that the patient may have consumed dairy products contaminated with *L. monocytogenes*, which entered the bloodstream via the gastrointestinal tract and then spread to the hip joint. Therefore, the patient was diagnosed with listeriosis presenting as a late-onset prosthetic left hip infection.

**Figure 4:**
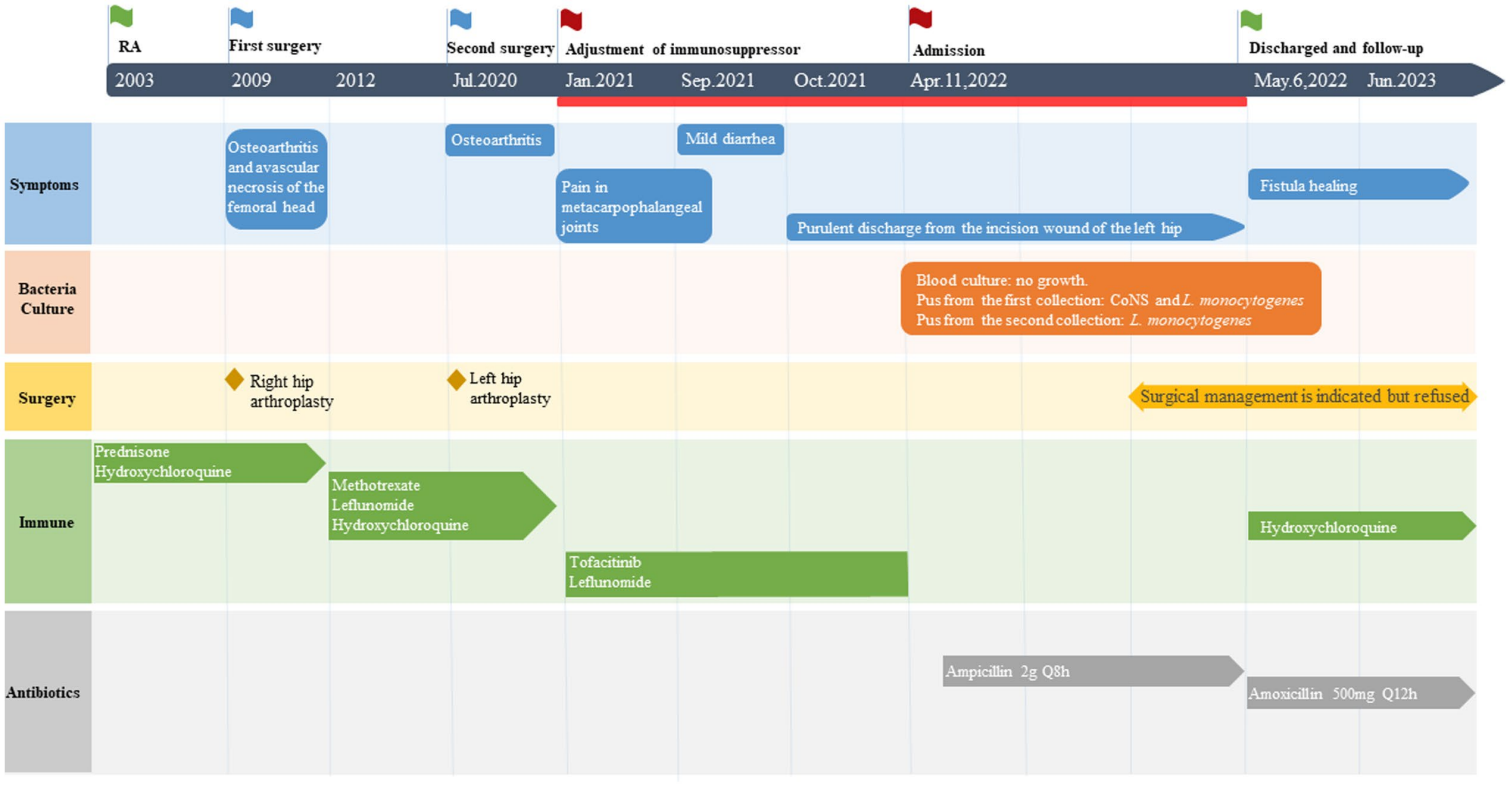
Timescale of the patient’s medical history.

The antimicrobial susceptibility testing of *L. monocytogenes* showed penicillin minimum inhibitory concentration of 0.19 μg/mL. The strain was susceptible to penicillin according to the CLSI standards. The zone size of trimethoprim-sulfamethoxazole, gentamicin, and meropenem using disk diffusion tests were all within the susceptible range. A renal-adjusted dose of intravenous infusion of ampicillin 2 g every 8 h was initiated on April 22, with regular dressing of the fistula site. The option of a combination of antibiotics with surgical management was offered to the patient, but it was refused by the patient. The antibiotics regimen was stepped down to life-long suppressive amoxicillin 500 mg every 12 h after 2 weeks of intravenous antibiotics. The patient did not experience any side effects of antibiotics or relapse of infection during therapy (Figure 4). The fistula was reported to be completely healed during a telephone follow-up in June 2023 (Figure 1B).

## Discussion

*L. monocytogenes* is a Gram-positive facultative anaerobic, motile, narrow-zone beta-hemolytic, non-spore-forming short rod bacterium that grows well at low temperatures between 4 and 10°C. It is an important foodborne pathogen and responsible for listeriosis which can be a serious infection with high mortality and hospitalization rate (4). The primary mode of transmission of *L. monocytogenes* occurs through contaminated foods, such as meat products, seafood, unpasteurized dairy products including ice cream, raw food products, and ready-to-eat food. According to WHO statistics, its incidence varies from 0.1 to 10 cases per one million people depending on the country or region (5). The China Center for Food Safety Risk Assessment has conducted investigations on food products in 28 provinces. The average prevalence rate of *L. monocytogenes* was found to be 4.42%, with meat and poultry products having the highest prevalence rate of 8.91%. However, between 2011 and 2016, only 253 cases of invasive listeriosis were reported in 19 provinces. The overall case fatality rate was 25.7%, and there were no reported deaths among mothers and children (6). It is important to note that these figures may underestimate the actual situation. Listeriosis is not a notifiable disease in mainland China and is not included in the national foodborne disease surveillance system. Therefore, the reported data may only represent a fraction of the total cases.

Although most healthy individuals infected do not develop symptoms since *L. monocytogenes* is an opportunistic pathogen but approximately 5 to 10% may become carriers who continue to shed bacteria via their feces. However, in immunocompromised people, *L. monocytogenes* can penetrate the intestinal mucosa and replicate within liver cells, then spread through the bloodstream to cause systemic infections (7). Cases of *L. monocytogenes* infections in artificial joints are rare, accounting for less than 2% of all artificial joint infections (8–10). In a retrospective study of 43 cases, it was found that *L. monocytogenes* joint and bone infections were mainly found in the prosthetic joints (79%, 34/43), with the median time of infection after insertion being 9 years (0.1–22). The infections were more likely to occur in patients on immunosuppressants in the past 5 years (33%, 14/43) (11). In another literature review of case reports, prosthetic joint infections due to *L. monocytogenes* mainly affected the hip (70%, 48/68) and knee (30%, 20/68) joints, and about 31% of the patients were on immunosuppressants. *L. monocytogenes* infections in prosthetic joints can manifest months to years after surgery, with an average onset time of 6.8 years, ranging from 2 months to 21 years (12). Among them, there were 7 and 10 patients with rheumatoid arthritis in the two articles, respectively. Coincidentally, the patient reported here, who had rheumatoid arthritis on immunosuppressive agents, developed an infection 16 months after surgery. This suggests that patients with rheumatoid arthritis who have undergone joint replacement, especially with concurrent use of immunosuppressive agents, are at high risk of listeriosis.

*L. monocytogenes* is an intracellular facultative pathogen, and the human immune response to it mainly depends on adaptive immunity mediated by T lymphocytes induced by dendritic cells. These T lymphocytes secrete IL-18, interferon-γ, perforin, and lysozyme, which stimulate macrophages to produce tumor necrosis factor and nitric oxide and to perform phagocytosis. Nevertheless, it is important to note that *L. monocytogenes* secretes Listeriolysin O, a pore-forming toxin. This toxin can induce T cell receptor unresponsiveness by promoting the expression of negative regulators of the T cell receptor signaling pathway, thereby suppressing antigen-induced T cell activation and impairing the posttranslational modification of host cell proteins. Finally, the interference with the host immune response leads to the development of listeriosis (13). Leflunomide is a non-biological disease-modifying antirheumatic drug (DMARD), which is a selective inhibitor of *de novo* pyrimidine synthesis. It works by inhibiting the growth of immune cells, including those contributing to the inflammatory response. While it is beneficial for managing autoimmune disease symptoms, it can also reduce the body’s ability to fight off infections. Common infections associated with leflunomide use include respiratory tract infections, urinary tract infections, as well as skin and soft tissue infections. However, there have been no reported cases of prosthetic joint infection with leflunomide. On the other hand, tofacitinib, as a JAK inhibitor, can theoretically inhibit the development and/or maintenance of pathogen-specific memory T cells by inhibiting the intracellular signaling of IL-12, interferon-γ, and other related cytokines (3). Opportunistic infections, including tuberculosis, *Pneumocystis carinii* pneumonia, and cryptococcosis, have been proposed to be associated with the use of JAK inhibitors, but the mechanisms have not been elucidated. It is hypothesized that tofacitinib may disrupt the normal functioning of key components involved in adaptive immunity, thereby impacting its overall effectiveness (14). It has been proposed that the transcriptional response to *L. monocytogenes* infection requires a cooperative signal of type I interferon (IFN-I) -stimulated JAK–STAT and proinflammatory NF-κB pathways. In other words, if there is inhibition of the JAK–STAT pathway, then the transcriptional response to *Listeria* infection may be impaired and listeriosis may be more likely to develop (15). Before this, we summarized the types of targeted therapies and *Listeria* infection, including Anti-TNF-α monoclonal antibodies, Anti-CD52 monoclonal antibodies, Anti-CD20 monoclonal antibodies, Anti-IL-6 receptor monoclonal antibody, EGFR/HER2 pathway inhibitors, Anti-CD38 monoclonal antibody and protease inhibitors. Most cases of *Listeria* infection have been associated with the use of anti-TNF-α therapy (16). However, in an open-label randomized controlled trial involving rheumatoid arthritis patients on Tofacitinib therapy, the risk of opportunistic infections was found to be even higher than that observed in patients receiving anti-TNF-α therapy (17).

Based on these mechanisms and observed phenomena, it is suggested that the use of JAK inhibitors in patients with rheumatoid arthritis may be associated with an increased risk of occult listeriosis. Therefore, under the guidance of her rheumatologist, the patient discontinued tofacitinib to appropriately reduce her immunosuppression and achieve stable control of listeriosis as soon as possible.

Positive regulatory factor A (PrfA) is a key regulator of *L. monocytogenes* pathogenesis and induces the expression of multiple virulence factors within the infected host. Furthermore, prfA plays an important role in the ability to form biofilms, which shown to affect the expression of 175 genes during biofilm formation compared to a wild type and a prfA deletion mutant. The mutation of the poison force regulating factor, *prf*A gene, can affect the biofilm formation (18–20). As *L. monocytogenes* tend to form biofilms on materials such as titanium used for joint implants, it can survive and grow extensively over a prolonged period of time (18). Hence, after invading the body and migrating toward the implant site, *L. monocytogenes* poses a significant risk by potentially causing insidious onset or slow progression of infection which may lead to irreversible tissue damage if left untreated or misdiagnosed. In this patient’s case, we speculate that a mutation in the *prf*A gene may have been present in *L. monocytogenes*, which invaded the gut and migrated to the left hip prosthesis, followed by biofilm formation and insidious growth, leading to pyogenic arthritis.

Although there are no guidelines or expert consensus on the diagnosis and treatment of *L. monocytogenes* joint implant infections, we believe that scientific and standardized strategies are needed for the management of such diseases. Research has proposed that the antibiotic treatment regimen for *L. monocytogenes* purulent arthritis ranges from parenteral administration for 2 weeks to oral administration for 6 months, combined with surgical treatment such as debridement, removal of implants, or joint fusion (12). Because *L. monocytogenes* is intrinsically resistant to all generation of cephalosporin, antibiotic selection is particularly important. The current preferred antibiotic treatment for infection is beta-lactam antibiotics, usually ampicillin or amoxicillin, and when combined with intracranial infection, gentamicin is used to enhance bactericidal activity. Trimethoprim-sulfamethoxazole is the main alternative to penicillin for patients with penicillin allergy. Meropenem and linezolid can be used in patients who cannot tolerate penicillin or trimethoprim-sulfamethoxazole.

## Conclusion

In summary, patients with rheumatoid arthritis who are treated with tofacitinib might have an increased chance of developing a prosthetic joint infection caused by *L. monocytogenes.* Joint replacement surgery is frequently used to address complications of rheumatoid arthritis, such as osteoarthritis and femoral head necrosis. The use of targeted therapy, such as tofacitinib, may potentially increase the risk of opportunistic infections among these patients. If patients present with symptoms such as unexplained fever, joint pain, or swelling in the joint implant, it would be important to consider the possibility of an *L. monocytogenes* infection. Upon diagnosis, a treatment regimen typically involving beta-lactam antibiotics and surgical intervention may be required. Furthermore, it is imperative for high-risk patients to adhere to healthcare professionals’ advice regarding the avoidance of food that poses a risk for listeriosis.

## Data availability statement

The original contributions presented in the study are included in the article/supplementary material, further inquiries can be directed to the corresponding author.

## Ethics statement

The studies involving humans were approved by The Ethics Committee of the University of Hong Kong-Shenzhen Hospital. The studies were conducted in accordance with the local legislation and institutional requirements. The human samples used in this study were acquired from a by- product of routine care or industry. Written informed consent for participation was not required from the participants or the participants' legal guardians/next of kin in accordance with the national legislation and institutional requirements. Written informed consent was obtained from the individual(s) for the publication of any potentially identifiable images or data included in this article.

## Author contributions

CD: Conceptualization, Data curation, Investigation, Writing – original draft, Writing – review & editing. KC: Conceptualization, Supervision, Writing – review & editing. NL: Data curation, Writing – review & editing. FX: Conceptualization, Data curation, Supervision, Writing – review & editing.
